# Therapeutic potential of salidroside in preserving rat cochlea organ of corti from gentamicin-induced injury through modulation of NRF2 signaling and GSK3β/NF-κB pathway

**DOI:** 10.1371/journal.pone.0298529

**Published:** 2024-03-14

**Authors:** Yan Zhang, Shuyuan Yu, Xinyi Guo, Luoying Wang, Ling Yu, Ping Wang

**Affiliations:** 1 Department of Otolaryngology-Head and Neck Surgery, The First Hospital of Jilin University, Changchun, Jilin, China; 2 Department of Regenerative Medicine, College of Pharmacy, Jilin University, Changchun, Jilin, China; 3 Department of Pharmacy, the Second Hospital of Jilin University, Changchun, Jilin, China; Penn State Health Milton S Hershey Medical Center, UNITED STATES

## Abstract

Salidroside (SAL) is a phenol glycoside compound found in plants of the *Rhodiola* genus which has natural antioxidant and free radical scavenging properties. SAL are able to protect against manganese-induced ototoxicity. However, the molecular mechanism by which SAL reduces levels of reactive oxygen species (ROS) is unclear. Here, we established an *in vitro* gentamicin (GM) ototoxicity model to observe the protective effect of SAL on GM-induced hair cells (HC) damage. Cochlear explants of postnatal day 4 rats were obtained and randomly divided into six groups: two model groups (treatment with 0.2 mM or 0.4 mM GM for 24 h); two 400 μmol/L SAL-pretreated groups pretreatment with SAL for 3 h followed by GM treatment (0.2 mM or 0.4 mM) for 24 h; 400 μmol/L SAL group (treatment with SAL for 24 h); control group (normal cultured cochlear explants). The protective effects of SAL on GM-induced HC damage, and on mRNA and protein levels of antioxidant enzymes were observed. HC loss occurred after 24 h of GM treatment. Pretreatment with SAL significantly reduced GM-induced OHC loss. In cochlear tissues, mRNA and protein levels of NRF2 and HO-1 were enhanced in the GM alone group compared with the SAL pretreatment GM treatment group. SAL may protect against GM-induced ototoxicity by regulating the antioxidant defense system of cochlear tissues; SAL can activate NRF2/HO-1 signaling, inhibit NF-κB activation, activate AKT, and increase inhibitory phosphorylation of GSK3β to decrease GSK3 activity, all of which exert antioxidant effects.

## 1. Introduction

Aminoglycosides are broad-spectrum antibiotics that are widely used for the treatment of acute, severe infections, and recurrent respiratory tract infections. They have good therapeutic efficacy, however their application is limited due to the induced severe nephro-, oto- and vestibular toxicity [1]. Kidney injury caused by gentamicin (GM) is usually reversible by early detection and ceasing administration because proximal renal tubular cells are able to regenerate [2]. However, damage to mammalian cochlear hair cells (HCs) caused by GM can be more consequential because mammalian cochlear HCs do not regenerate under normal conditions [3]. Hair cells have a mechanotransduction function and possess many cytoplasmic mitochondria. Hearing loss caused by noise, age, ototoxic drugs and cochlear ischemia-reperfusion injury are usually associated with reactive oxygen species (ROS)-induced oxidative stress [4–7]. Aminoglycoside antibiotics target bacteria can also cause mitochondrial dysfunction, induce the release of pro-apoptotic factors and oxidases into the cytoplasm and generate free radicals [8]. The primary cause of GM ototoxicity is the oxygen free radicals’ production, which can lead damage to cells through a series of peroxidation reactions, and reduced peroxidation of unsaturated fatty acids in phospholipid molecules in the cell membrane [9]. Reducing GM-induced ROS production is a strategy for the treatment and prevention of aminoglycoside ototoxicity.

Salidroside (SAL) is a phenol glycoside compound which found in *Rhodiola* genus plants and able to extracted from the roots and stems of the plant or synthesized using chemical and biological methods. SAL has natural antioxidant effects and multiple biological activities, including protection of the cardiovascular system, ameliorates memory impairment, anti-tumor and anti-asthmatic effects [10–14]. Zhang et al. found that SAL protected primary cultured rat cortical neurons from cobalt chloride-induced hypoxic injury in a dose-dependent manner. This protective effect may occur through increased neuronal HIF1 expression, decreased intracellular ROS activity, and reduced overexpression of NF-κB protein [15]. SAL can also activate the NRF2 signaling pathway, promote the nuclear translocation of NRF2, and reduce oxidative stress levels by increasing the expression of downstream antioxidant enzymes, thereby reducing oxygen free radical-induced damage in the heart [16]. SAL can protect human umbilical vein endothelial cells (HUVECs) from ROS damage by regulating REDD1/mTOR signaling [17], which can also exert a protective effect on nerve cells by activating RhoA-MAPK and PI3K/AKT signaling, inhibiting inflammatory cytokine expression, and regulating the PI3K/AKT pathway [18]. SAL induces directional differentiation of mesenchymal stem cells (MSCs) into neuronal cells by inhibiting Notch signaling and activating BMP signaling [19], and affects the directional differentiation of neural cells by altering gene expression in the cAMP/Ca^2+^ signaling pathway [20].

There are few reports on the effects of SAL on the auditory system. Ding et al. found that 1–10 μM SAL can protect cochlear HCs and neurons from manganese-induced damage, which indicates that SAL can inhibit cochlear HC apoptosis [21]. However, the effect of SAL on GM-induced ototoxicity has not been reported. Here, the cochlear explants were cultured *in vitro*, observed the protective effect of SAL on GM-induced HC loss, and explored the possible molecular mechanism for the antioxidant effect of SAL by treating cells with different signaling pathway inhibitors.

## 2. Materials and methods

### 2.1 Animals

Eighteen-day pregnant Wistar rats were purchased from the Animal Centre of Jilin University (certificate number SYXK (JI) 2016–0001). This study was approved by the Ethics Committee of Jilin University (Ethical Approval Number 2018–101), and the experimental procedures were in accordance with the institutional guidelines of the Animal Care and Use Committee of Jilin University. The rats were breed in cages designed to permit *ad libitum* access to chow and water in rooms with a light-dark cycle. The room temperature was 23 ± 1°C and humidity was 40 ± 5%. The study was performed using 4-day-old pups delivered by the pregnant dams.

### 2.2 Isolation and culture of cochlear explant

Postnatal-4-day rats were sacrificed by CO2 anesthesia [22], then decapitated under sterile conditions and the cochlea were removed and placed into cold D-Hank’s solution. The spiral ligament was carefully dissected from each otic capsule, leaving the whole explant with spiral ganglion neurons. Five hundred microliters of complete culture medium (DMEM/F12+10% FBS+100 U penicillin) were added to a 35 mm culture dish and incubated at room temperature for 30 min. An isolated explant in a natural curved shape was then placed at the bottom of the culture dish and incubated for 3 h. Then, 1 ml of complete medium was added to culture dish, and culture continued at 37°C, in 5% CO_2_, and saturated humidity [23].

### 2.3 Grouping

To determine the protective effect of SAL (and its effective dose) against GM-induced HC damage, cochlear explants were isolated and cultured with SAL at different concentrations for 24 h. HCs were then stained, and the effect of SAL on HC survival was observed.

The cochlear explants were randomly divided into six groups: two model groups (cochlear explants cultured with 0.2 mM or 0.4 mM GM for 24 h), two 0.4 mM/LSAL-pretreated groups [cochlear explants pretreated with SAL for 3 h followed by of GM treatment (0.2 mM or 0.4 mM) for 24 h], 0.4 mM/l SAL group (cochlear explants treated with SAL for 27h), control group (normal cultured cochlear explants). The protective effects of SAL on GM-induced HC damage, and on mRNA and protein levels of antioxidant enzymes were observed. Signaling pathway inhibitors, including the NRf2 inhibitor, trigonelline (10 μmol/L), the NF-κB inhibitor, PDTC (10 μmol/L), the PI3K/AKT inhibitor, LY294002 (1.0 μmol/L), and the GSK3β inhibitor, AR-A014418 (10μmol/L), were added to culture medium at the same time as GM.

### 2.4 Staining cells with TRITC-labeled phalloidin

Following the culture process, the medium was discarded and the specimens were fixed with 4% paraformaldehyde for 30 min, washed three times with PBS, and then incubated with 1% Triton X-100 for 20 min. Cells were stained with TRITC-labeled phalloidin (1:200) at room temperature for 20 min. Specimens were then mounted with glycerin, observed and photographed under the confocal scanning laser microscope. The number of HCs was counted from five randomly selected fields of the basal/mid-basal cochlear turns (200 μm each), and the average number of cells in the randomly selected fields were calculated.

### 2.5 Detection of malondialdehyde content and superoxide dismutase and glutathione peroxidase activities

Malondialdehyde (MDA) content was determined using thiobarbituric acid, superoxide dismutase (SOD) activity was detected by using xanthine oxidase, and glutathione peroxidase (GSHPx) activity was detected by using 5–5’-dithiobis-(2-nitrobenzoic acid) (DTNB), as previously described [24]. Homogenates (10%) were prepared by disrupting cells and supernatants were collected after centrifugation. Absorbance was measured using an ultraviolet spectrophotometer. MDA, SOD, GSHPx activities were calculated.

### 2.6 RNA isolation and quantitative real-time PCR

Total RNA from cultured cochlear explants was extracted using Trizol solution (Life Technologies, USA). cDNA was synthesized using a first strand cDNA Synthesis kit according to the manufacturer’s instructions. Real time PCR was performed using the FastStart Universal SYBRGreen Master kit. The PCR protocol comprised an initial denaturation at 95°C for 5 min; 35 cycles of denaturation at 94°C for 10s, annealing at 55°C for 10 s, and elongation at 72°C for 15 s; and then a final elongation at 72°C for 10 min. Fluorescence values were collected during the reaction. The specificity of the amplicon was determined by melting curve analysis. *Ho-1* as the downstream molecule of *Nrf2* [25]. The target gene level was normalized against that of *Gapdh* mRNA and relative values for *Nrf2* and *Ho-1* mRNA levels were calculated using the formula: fold change = 2^−ΔΔCT^.

### 2.7 Western blot analysis

Cochlear explants were lysed with RIPA buffer containing a protein phosphatase inhibitor cocktail (Roche Applied Science, Germany) and centrifuged for 10 min at 12000 rpm. Protein concentrations of supernatants were determined using a BCA assay kit (Beyotime Institute of Biotechnology, Shanghai, China) and then equal amounts of protein were denatured by heating for 10 min at 90°C after adding 5×SDS-PAGE loading buffer. Proteins were then separated by 10% SDS-PAGE electrophoresis and transferred to PVDF membranes. The membranes were blocked in TTBS containing 5% BSA for 1 h and then incubated with different primary antibodies (AKT, 1:5000, phospho-AKT, 1:500, GSK3β,1:1000, phospho-GSK3β,1:2000, NF-κB p65 (Ser536), 1:1000; phospho-p65, 1:500) in TTBS containing 3% BSA overnight at 4°C. Membranes were then incubated with an HRP-labeled secondary antibody at room temperature for 2 h, and then protein bands were visualize using a SuperSignal West Dura Extended Duration Substrate kit (Thermo Fisher Scientific, Waltham, MA, USA). Images were obtained using a Dolphin‑C image system (Wealtec Corp., Sparks, NV, USA). Immunoblots were quantified using the gray values for each protein band using Image J software. Relative protein levels were calculated using β-actin as an internal standard. All experiments were performed in triplicate.

### 2.8 Statistical analysis

All data were collected from at least three experimental groups and are expressed as the mean ± SD. Data were analyzed using GraphPad Prism version 8.0 (GraphPad software, Inc., USA). Statistical analysis involved the two-tailed unpaired Student’s t-test, the Mann-Whitney test, and one-way analysis of variance (ANOVA) with post hoc analysis by Tukey’s multiple-comparison test as indicated. P < 0.05 was considered a statistically significant difference.

## 3. Results

### 3.1 SAL at low concentration is not toxic to cochlear HCs cultured in vitro

**Fig 1:**
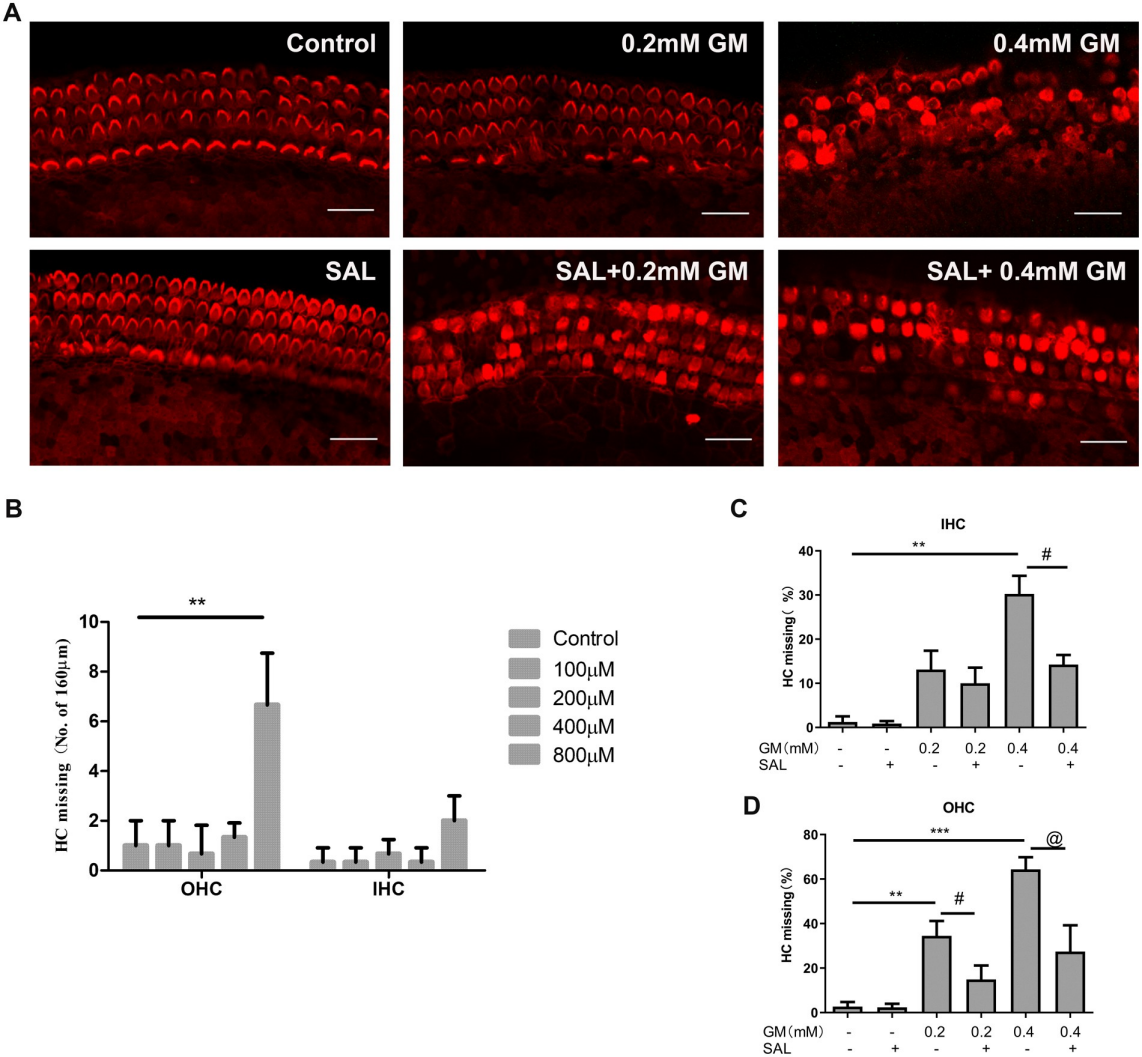
SAL reduces GM-induced cochlear hair cell loss. A: Cochlear explants were pretreated with 0.4 mM SAL followed by GM treatment. After 24 hours of combination treatment, stained with TRITC-labeled phalloidin, and the red fluorescence indicated F-actin-labeled HC stereocilia. Morphological observation of inner and outer hair cells under confocal laser scanning microscopy. Scale bar, 20 μm. C,D: The percentage of hair cell loss was counted from five selected fields of basal/mid-basal cochlear turns. GraphPad Prism was used to analyze differences in hair cell survival in each group. ***p<0.001, **p<0.01 compared with the control group, #, @ p<0.05 compared with the GM group. B: outer hair cells, C: inner hair cells. SAL: salidroside, GM1: 0.2 mM gentamicin, GM2: 0.4 mM gentamicin. The white arrows indicate hair cells, OHC: outer hair cells, IHC: inner hair cells. B: Changes in cochlear hair cell loss after treatment with different concentrations of SAL. Cochlear explants were treated with 0.1, 0.2, 0.4, 0.8 mM SAL for 24 h, and the stereocilia of HC were stained with TRITC-labeled phalloidin. IHC and OHC loss from five randomly selected fields of the basal/mid-basal cochlear turns (200 μm each) were counted. SAL at 0.8 mM caused occasional loss of OHCs. **p<0.01 compared with the control group.

SAL exerts a neuroprotective effect *in vitro* at concentrations ranging from 1.0 μM to 1 mM, and the most commonly used experimental concentration is 0.1 mM [26]. *In vitro* culture of the cochlear explants is different from monolayer cell culture. Therefore, in this study, we used 0.1, 0.2, 0.4, 0.8 mM SAL to treat cochlear tissues for 24 h. HCs were stained for F-actin, and HC loss was determined. As shown in Fig 1B, 0.1 mM—0.4 mM SAL did not cause obvious damage to cochlear HCs. A slight increase in outer hair cell (OHC) loss (approximately 7.5%) was observed with 0.8 mM SAL.

### 3.2 SAL pretreatment effectively reduces GM-induced cochlear HC loss

Cochlear explants were pre-treated with 0.4 mM SAL for 3 h and then treated with 0.2 mM or 0.4 mM GM for 24 h. Five fields of basal/mid-basal cochlear turns were selected, and the HC loss rate determined. The OHC loss rate was 38% after 0.2 mM GM treatment, and 68% after 0.4 mM GM treatment. The OHC loss rates were 16% and 34%, after SAL pretreatment combined with 0.2 mM and 0.4 mM GM treatment for 24 h, respectively. SAL pretreatment combined with GM significantly decreased OHC loss rate compared with GM alone (Fig 1A, SAL group). The IHC loss rate in the 0.2 mM GM treatment group was higher than that in the SAL+GM group, but this difference was not statistically significant. The IHC loss rate in the 0.4 mM GM treatment group was significantly higher than that of the SAL+GM group (p <0.05, Fig 1A, 0.4mM GM group).

### 3.3 SAL enhances activities of antioxidant enzymes in cochlear tissues

**Fig 2:**
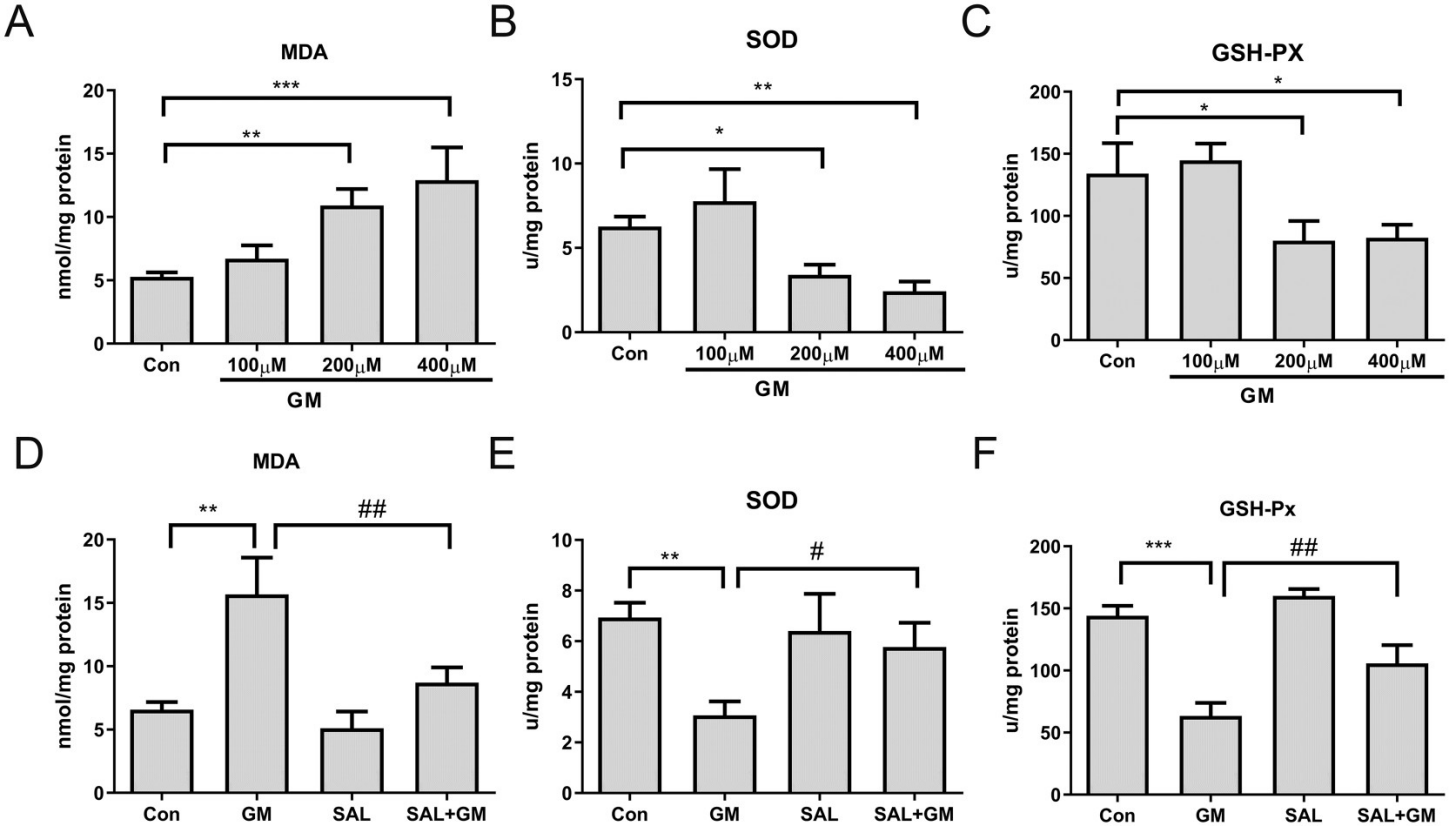
Effect of GM and SAL on superoxide dismutase (SOD), GSHPx, and malondialdehyde (MDA) concentrations in cochlear tissues cultured in vitro. A-C: Changes in SOD, GSHPx and MDA concentrations after GM treatment. ***p<0.001, **p<0.01, *p<0.05 versus control group. D–E: Effect of SAL on SOD, GSHPx and MDA concentrations in the GM-induced ototoxicity model. ***p<0.001, **p<0.01 versus control, ##p<0.01, #p<0.05 versus GM group. SAL: salidroside, GM: 0.4 mM gentamicin.

Changes in the level of the oxidative stress marker, MDA, and in the activities of antioxidant enzymes, SOD and GSHPx, in cochlear tissue were detected using thiobarbituric acid, xanthine oxidase, and 5–5’-dithiobis-(2-nitrobenzoic acid), respectively. The MDA content in cochlear tissues increased in a dose-dependent manner with increasing concentration of GM. SOD activity showed an increasing trend after treatment with 0.1 mM GM, but SOD activity was significantly decreased after treatment with 0.2 mM and 0.4 mM GM (both p<0.05). GSHPx activity showed a similar trend to that of SOD activity (both p<0.05, Fig 2A–2C). After 0.4 mM SAL pretreatment followed by 0.4 mM GM treatment, the MDA content was decreased and the activities of SOD and GSHPx were increased in cochlear tissues (Fig 2D–2F). These results indicate that treatment with GM may lead to an increased oxidative stress levels in isolated cochlear tissues, and that SAL exerts an opposite regulatory effect on oxidative stress levels.

### 3.4 SAL up-regulates NRF2/HO-1 activity in cochlear tissue

**Fig 3:**
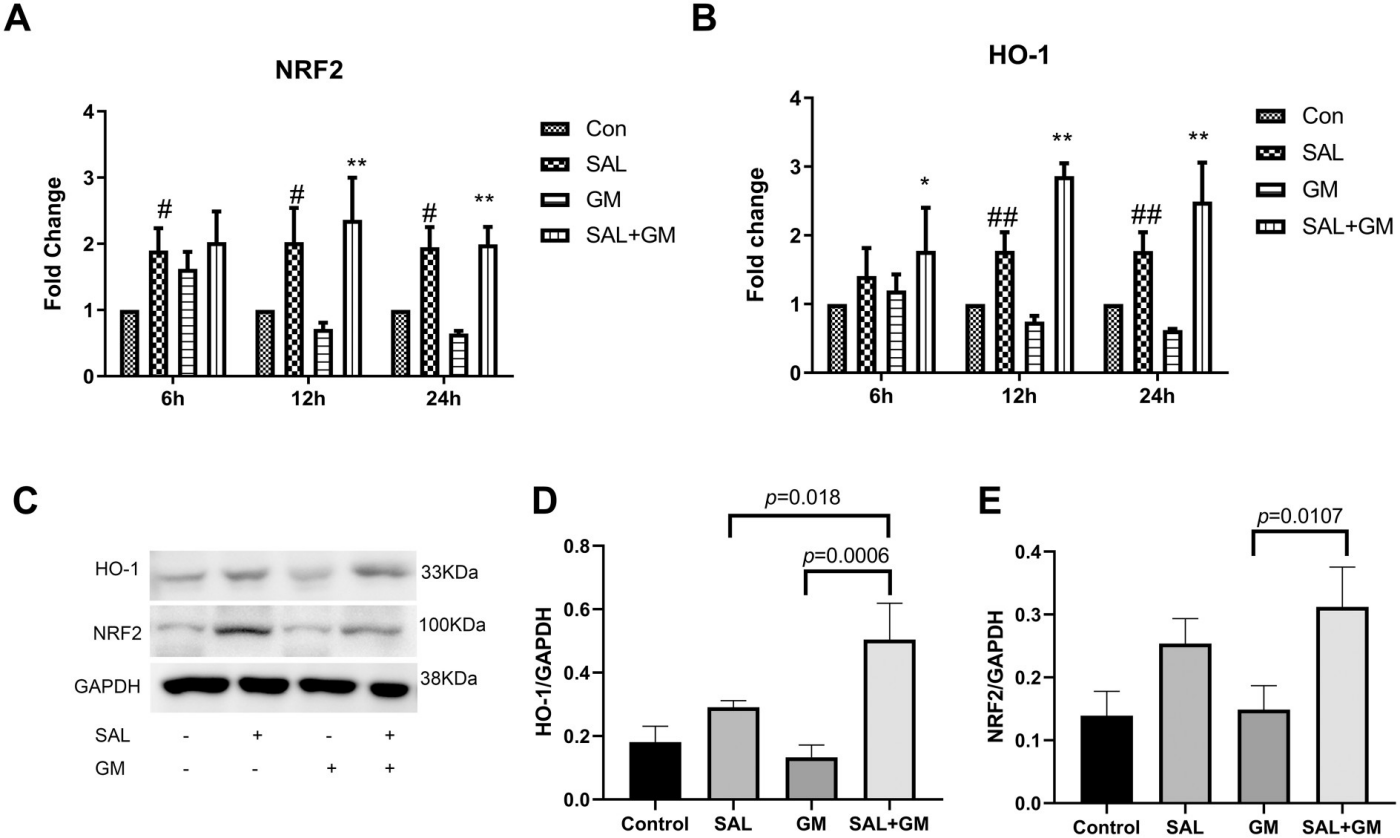
Effects of SAL on the mRNA and protein levels of NRF2 and HO-1 in cochlear tissues after GM treatment in vitro. A: GM administration changed the Nrf2 mRNA level with time. B: GM administration changed the Ho-1 mRNA level with time. **p<0.01, *p<0.05 versus GM group, ##p<0.01, #p<0.05 versus control group. C: Representative image of a NRF2 and HO-1 western blot of untreated, GM, SAL, and SAL+GM treated groups. D: Optical density image analysis of NRF2 protein. E: Optical density image analysis of HO-1 protein. All data were normalized to GAPDH.

ROS produced by oxidative stress directly or indirectly destroys macromolecules in cells. However, cells possess a complex antioxidant defense system that protects against oxidative stress-induced cytotoxicity by increasing the expression of antioxidant-related proteins. NRF2 is a key factor involved in cellular responses to oxidative stress. Here, qPCR and western blot analysis were used to detect the mRNA and protein levels of NRF2 and its downstream target, HO-1. Fig 3A and 3B shows the change in *Nrf2* and *Ho-1* mRNA levels after GM and SAL treatment. The mRNA level of *Nrf2* was significantly reduced after 12 and 24 hours of GM treatment compared with the SAL +GM group (both p<0.01). The mRNA level of *HO-1* was also significantly reduced after 6, 12 and 24 hours of GM treatment compared with the SAL group (both p<0.01). SAL increased mRNA levels of *Nrf2* and *HO-1* in cochlear tissues treated with or without GM. Changes in protein levels after 12 hours of GM treatment were detected by western blot analysis, as shown in Fig 3C–3E. The protein level of NRF2 or HO-1 also showed an increasing trend in the SAL group, but there was no significant difference between SAL and control groups. SAL pretreatment combined with GM significantly increased protein levels of NRF2 (p<0.05) and HO-1 (p<0.01) in cochlear tissues compared with the GM alone group. These results indicate that under oxidative stress conditions, SAL exerts a protective effect against oxidative stress-induced damage in cochlear tissue by regulating the NRF2/HO-1 signaling pathway.

### 3.5 SAL activates AKT and inhibits GSK3β

**Fig 4:**
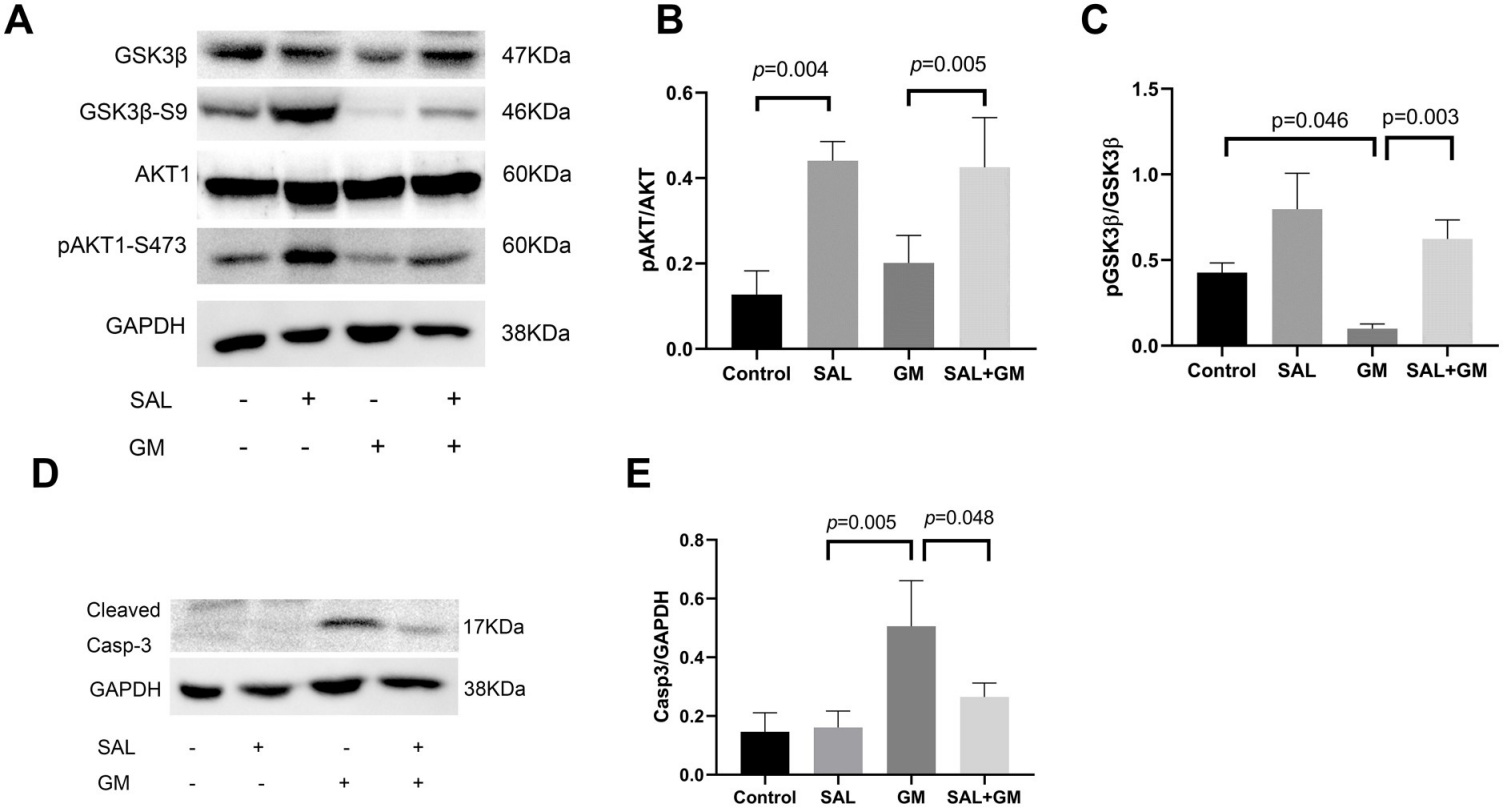
Effects of SAL on the protein levels of AKT, GSK3β and caspase 3 in cochlear tissues after GM treatment in vitro. A: Western blot image of AKT and GSK3β. B: The pAKT/AKT ratio was analyzed in different treatment groups using Image J software. C: The pGSK3β/GSK3β ratio was analyzed in different treatment groups. D: Western blot image of caspase 3. E: Optical density image analysis of caspase 3.

To further clarify the regulatory effect of SAL on NRF2/HO-1 signaling, we detected changes in AKT, GSK3β, and caspase 3 activities (Fig 4A–4E). GSK3β phosphorylation of serine 9 causes negative regulation, with GSK3β activity inhibited after phosphorylation. The level of pGSK3β(Ser9) was decreased in the GM-treated group compared with that in the control group (p<0.05), and the level of pAKT was also decreased in the GM-treated group. Interestingly, SAL pretreatment combined with GM significantly increased the level of pGSK3β(Ser9) and pAKT compared with the GM alone group (p<0.001). SAL pretreatment combined with GM also decreased cleaved caspase 3 activity compared with the GM treated group (p<0.05, Fig 4D and 4E).

### 3.6 Change of hair cells survival after Sal combined GM under different inhibitors treatment

**Fig 5:**
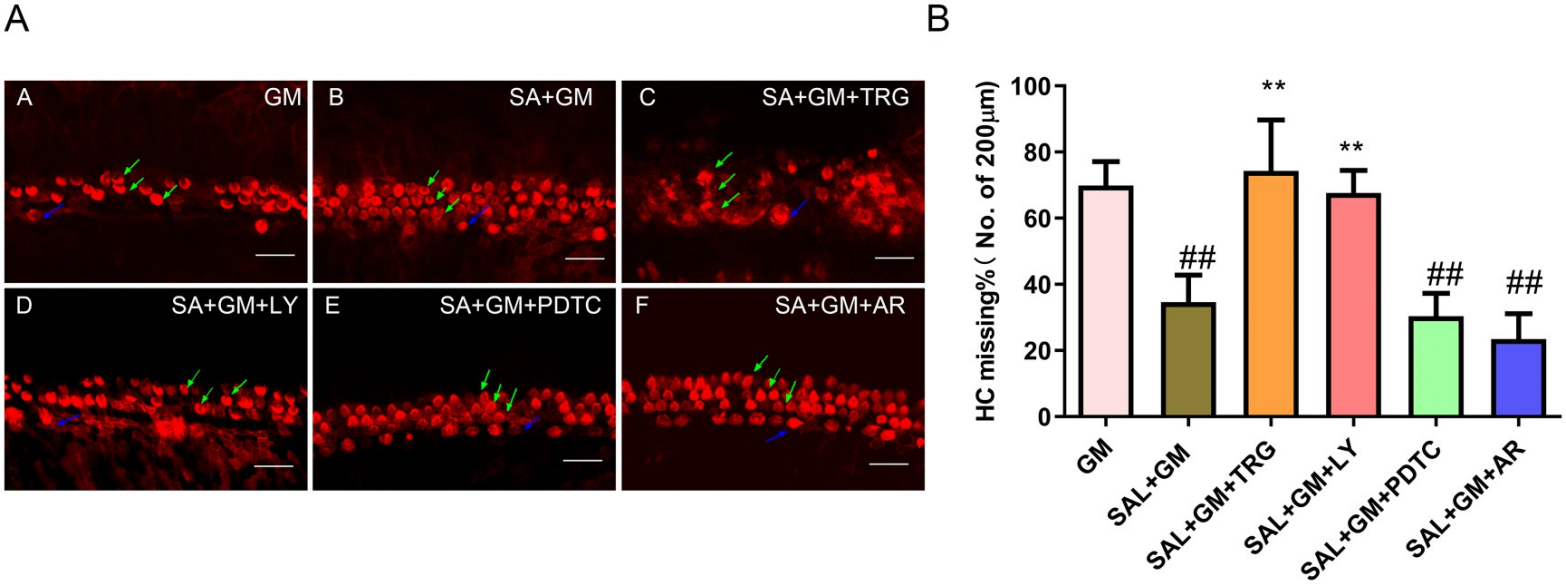
Cochlear HC loss in the SAL+GM group in response to different signaling pathway inhibitors. A: Confocal image of cochlear HCs treated with different inhibitors. Scale bar, 20 μm. The percentage of HC loss was counted from five selected fields of basal/mid-basal cochlear turns. B: GraphPad Prism was used to analyze differences in HC survival in each group. **p<0.01 versus SAL+GM group. ##p<0.01 versus GM group. The blue arrows indicate inner hair cells, and green arrows indicate outer hair cells.

Twenty-four hours after adding signaling pathway inhibitors to the SAL+GM group, the stereocilia of HCs were stained with TRITC-labeled phalloidin (Fig 5A). HC loss was counted in five fields of basal/mid-basal cochlear turns and the percentage of HC loss is shown in Fig 5B. In comparison with the SAL+GM group, the NRF2 inhibitor, trigonelline, and the PI3K/AKT inhibitor, LY294002, reversed the protective effect of SAL on GM-induced HC damage (p < 0.01). The protective effect of SAL was slight enhanced by addition of the GSK3β inhibitor, AR-A014418, but no significantly difference.

### 3.7 NRF2 expression change after inhibitor of LY294002 or AR-A014418 treatment

**Fig 6:**
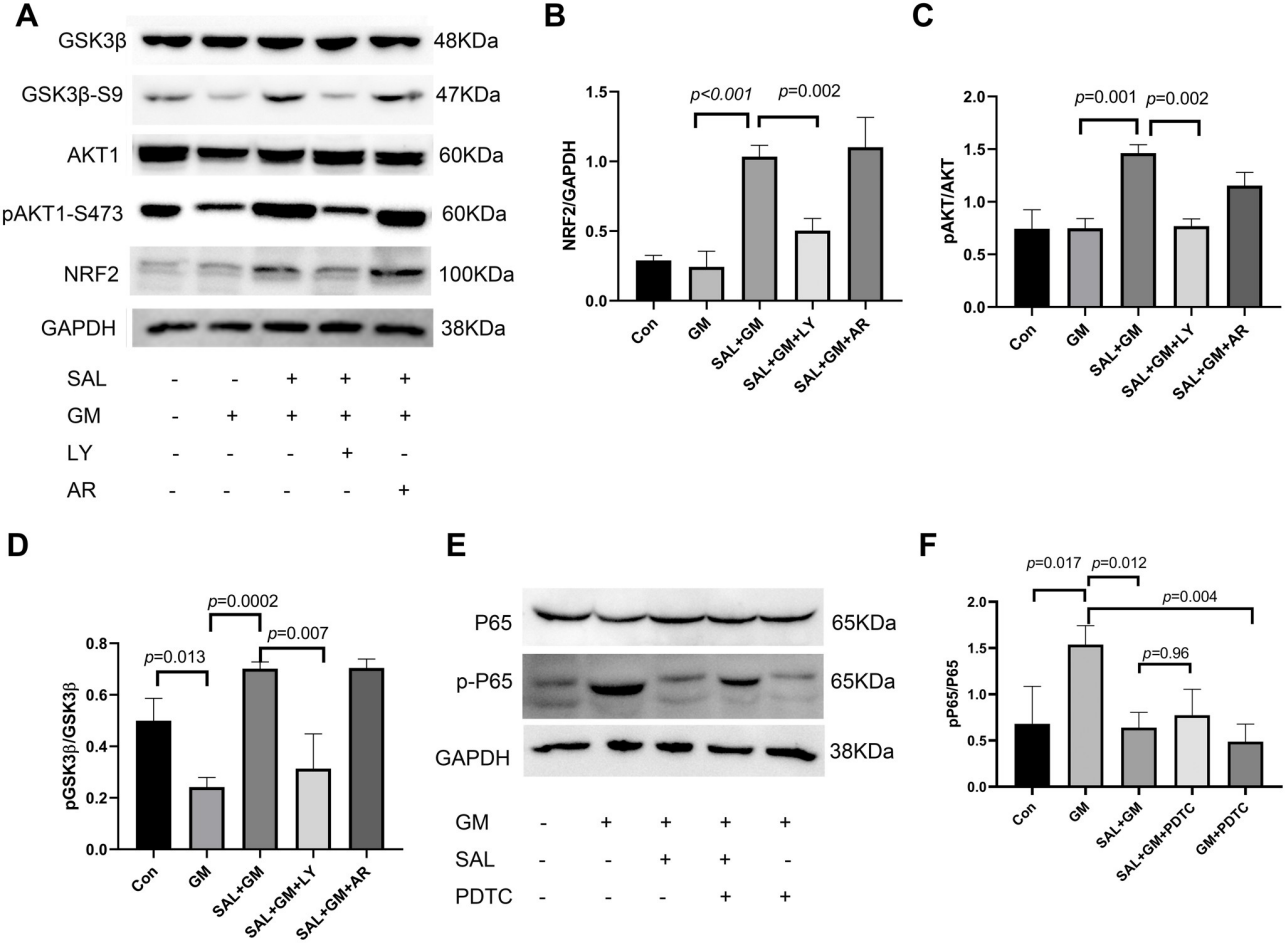
The expression of AKT, GSK3β, NF-κB and NRF2 in cochlear tissues in the SAL+GM group with or without signaling pathway inhibitors. A: Western blot image of AKT, NRF2 and GSK3β. B: Optical density image analysis of NRF2 protein. C: The pAKT/AKT ratio was analyzed in different treatment groups. D: The pGSK3β/GSK3β ratio was analyzed in different treatment groups. E: Western blot image of NF-κB in GM, SAL+GM, PDTC+GM and SAL+GM+PDTC-treated groups. F: The pNF-κB P65/NF-κB P65 ratio was analyzed in different treatment groups.

After adding signaling pathway inhibitors, the protein level of NRF2, and phosphorylation levels of AKT and GSK3β were detected by western blot analysis. The pAKT/AKT ratio was significantly increased in the SAL+GM group compared with the GM group (Fig 6A and 6C, p<0.01). In comparison with the SAL+GM group, the pAKT/AKT ratio was significantly decreased in the SAL+GM+ LY294002 group(p<0.01). There no significant differences between SAL+GM+ LY294002 and GM groups. The pGSK3β/GSK3β ratio was also significantly decreased in the SAL+GM+ LY294002 group compared with the SAL+GM group (Fig 6A and 6D, p<0.01). These results indicated that SAL increased PI3K/AKT levels and inhibited GSK3β activity. NRF2 protein levels were significantly increased in the SAL+GM group compared with the GM group (p<0.05). In comparison with SAL+GM group, NRF2 protein levels were significantly decreased in the SAL+GM+ LY294002 group (Fig 6A and 6B, p<0.01). NRF2 protein levels were increased in the SAL+GM+AR-A014418 group, but this increase was not significantly different from that in the SAL+GM group.

### 3.8 SAL inhibits GM-induced NF-κB activation

NF-κB requires signaling pathway to activate from the inactivation state in cytoplasm. The activating pathway of NF-κB were trigger by various of extracellular stimuli, which may result in phosphorylation of NF-κB inhibitor and subsequent proteasome-mediated degradation [27]. Activated NF-κB migrates into the nucleus to regulate the expression of multiple target genes. NF-κB inhibitor, PDTC, or SAL treatment combined with GM both down-regulated the phosphorylation of NF-κB p65 in the GM-treated group. However, there is no significant difference presented in the NF-κB p65 phosphorylation level between SAL+GM+PDTC and SAL+GM groups (Fig 6E and 6F), morphological results also supported the finding. The HC loss rate did not change in the SAL+GM group after addition of PDTC (Fig 5A). These results indicate that NF-κB is involved in the protective effect of SAL on GM-induced cochlear HC damage, and that in the protective effect of SAL on cochlear HCs, NF-κB may function as a downstream target of GSK3β.

## 4. Discussion

In this study, after the treatment of 0.2μM GM, the loss rate of OHC is 38%, while the OHC loss rate increase to 70% with 0.4mM GM treated. The concentration of MDA was increased with the concentration of GM in dose-depend manner in cochlear. SOD and GSH-Px were up-regulated with 100mM GM added. However, the SOD activity was significantly down-regulated with 200μM and 400μM GM added. mM and 0.4 mM GM increased the rate of OHC loss, also increased MDA content in cochlear tissues in a dose-dependent manner. SOD and GSHPx were increased by 0.1 mM GM, while 0.2 mM and 0.4 mM GM decreased SOD activity. Through the result, we speculate that GM at low concentrations may stimulate the intrinsic antioxidant defense system in cochlear tissues, while at high concentrations may cause excessive ROS production that overwhelms the antioxidant defense system.

As one of the most effective active ingredients in *Rhodiola rosea*, SAL is a phenolic glycoside obtained from the plant’s roots and rhizomes, it also attract many attention due to its own pharmacology and mechanism of action. SAL acts as an antioxidant in a wide range of concentrations, and the protects against ROS-induced cell damage. Direct incubation with 1 mM SAL produced neuroprotective effects in active neural stem cells [28], while *in vitro* culture of isolated cochlear explants with 1 μM SAL protects against Mn-induced damage to HCs and spiral ganglion neurons. In the current study, cochlear explants were treated with SAL at concentrations of 0.1 mM– 0.8 mM for 24 h. SAL concentrations of 0.1 Mm– 0.4 mM did not lead to damage to *in vitro* cultured cochlear HCs. However, occasional loss of OHCs was observed after treatment with 0.8 mM SAL. SAL at the concentration of 0.4 mMhad a significant protective effect against GM-induced HC damage. SAL also reduced MDA levels and increased activities of the antioxidant enzymes SOD and GSHPx in cochlear tissues. These findings directly demonstrate that SAL can protect against GM-induced HC damage by activating the antioxidant defense system.

Changes in the cellular redox state can trigger the expression of signal transduction factors that regulate the redox state. The transcription factor, NRF2, plays an important role in regulating the redox state of cells and the self-protective mechanism against oxidative damage. The NRF2/HO-1 signaling pathway is related to anti-oxidative stress and can scavenge ROS under conditions of oxidative stress. The KEAP1-NRF2-ARE pathway is associated with anti-apoptosis effects in animal models of noise-induced hearing loss [29]. In the central nervous system, SAL protects against inflammation-induced cognitive deficits by activating the SIRT1/NRF2 pathway [30]. SAL reduces HG-induced ROS generation and apoptosis and improves podocyte viability by upregulating HO-1 expression [31].

After the treatment of GM for 12 or 24 hours, the mRNA levels of *Nrf2* and *Ho-1* were reduced and indicated that activation of the NRF2/HO-1 signaling pathway is an adaptive response to stress. The increase in the level of *Nrf2* mRNA in the SAL+GM group was more obvious than that in the GM groups, indicating that under stress conditions, the regulation of NRF2 activation by SAL may be mediated via a feedback activation mechanism.

GM action on cochlear tissues can cause a large amount of ROS to be generated in the mitochondria of HCs, activation of caspase 3 and induction of apoptosis [32, 33]. SAL protects PC-12 cells by inhibiting Aβ 1-42-induced cytotoxicity and mitochondria-mediated endogenous caspase apoptotic pathways [34]. Our results show that the expression level of caspase 3 was increased after GM treatment but decreased after treatment with SAL. The protective effect of SAL on HCs is likely to be via an antioxidant effect, i.e. SAL reduces ROS generation by increasing the levels of SOD and GSH, which reduces caspase 3 expression.

To determine the molecular mechanism through which SAL exerts its protective effects, the PI3K/GSK3β pathway were choose due to it is an oxidative stress pathway that may be altered by GM toxicity. Oxidative stress leads to activation of GSK3β and reduction of NRF2 expression or nuclear translation in chlorpyrifos-induced brain toxicity after kaempferol administration [35]. Similarly, emodin treatment protects against oxygen-glucose deprivation/reoxygenation neurotoxicity by potentiating NRF2/ARE-regulated neuroprotection through the AMPK/GSK3β pathway [36]. Our results are compatible with previous studies indicating that cochlear HC loss after GM treatment is associated with oxidative stress. It is suggested that the protection of SAL against GM ototoxicity might be related to the activation of AKT and inhibition of GSK3β and modulation of NRF2/HO-1 signaling. NF-κB signaling regulated by GSK3β in nervous system, which reduces neuroinflammatory responses and oxidative stress in hippocampal neurons [37]. In a D-galactose-induced rat model of Alzheimer’s disease, SAL positively affected the inflammatory response and associated with the SIRT1/NF-κB signaling pathway [38]. This study further determined the relationship between the protective effect of SAL on GM-induced ototoxicity in cochlear HCs and its modulation of GSK3β and NRF2/HO-1 signaling pathways. Cochlear explants were treated with GM and SAL, and morphological changes were observed after treatment with trigonelline (an NRF2 inhibitor), AR-A014418 (a selective GSK3β inhibitor), PDTC (a NF-κB inhibitor) and LY294002 (a specific PI3-kinase inhibitor). Compared with the SAL+GM group, the percentage of HC loss was increased after trigonelline or LY294002 treatment. The percentage of HC loss was decreased after AR-A014418 treatment, but no difference was observed compared with the SAL+GM group. These results indicate that the AKT signaling pathway is involved in the protective effect of SAL on GM-induced ototoxicity, and the GSK3β and NF-κB may work as down-stream target of AKT regulation to be involved.

Further findings, SAL significantly increased levels of pGSK3β(Ser9) and pAKT(Ser273) compared with the GM alone group. After adding the GSK3β inhibitor, AR-A014418, to the SAL+GM group, the levels of pAKT(Ser273) and pGSK3β(SER9) did not change. However, after adding LY294002 to the SAL+GM group, the levels of pAKT(Ser273) and pGSK3β(Ser9) were significantly lower, indicating that SAL increases the expression of PI3K/AKT, which can increase the inhibitory activity of GSK3β. The PI3K/AKT signaling pathway acts as upstream of GSK3. SAL significantly increased NRF2 protein levels compared with the GM alone group, but the PI3K inhibitor, LY294002, significantly reduced the level of NRF2. NRF2 levels showed an increasing trend with the GSK3β inhibitor, AR-A014418, but there was no significant difference in NRF2 levels in the SAL+GM group before and after AR-A014418 treatment. We speculate that the SAL-induced AKT signaling pathway may directly regulate NRF2 expression. GSK3β signaling participates in the regulation of NRF2, although other molecular mechanisms may be involved in regulating NRF2.

**Fig 7:**
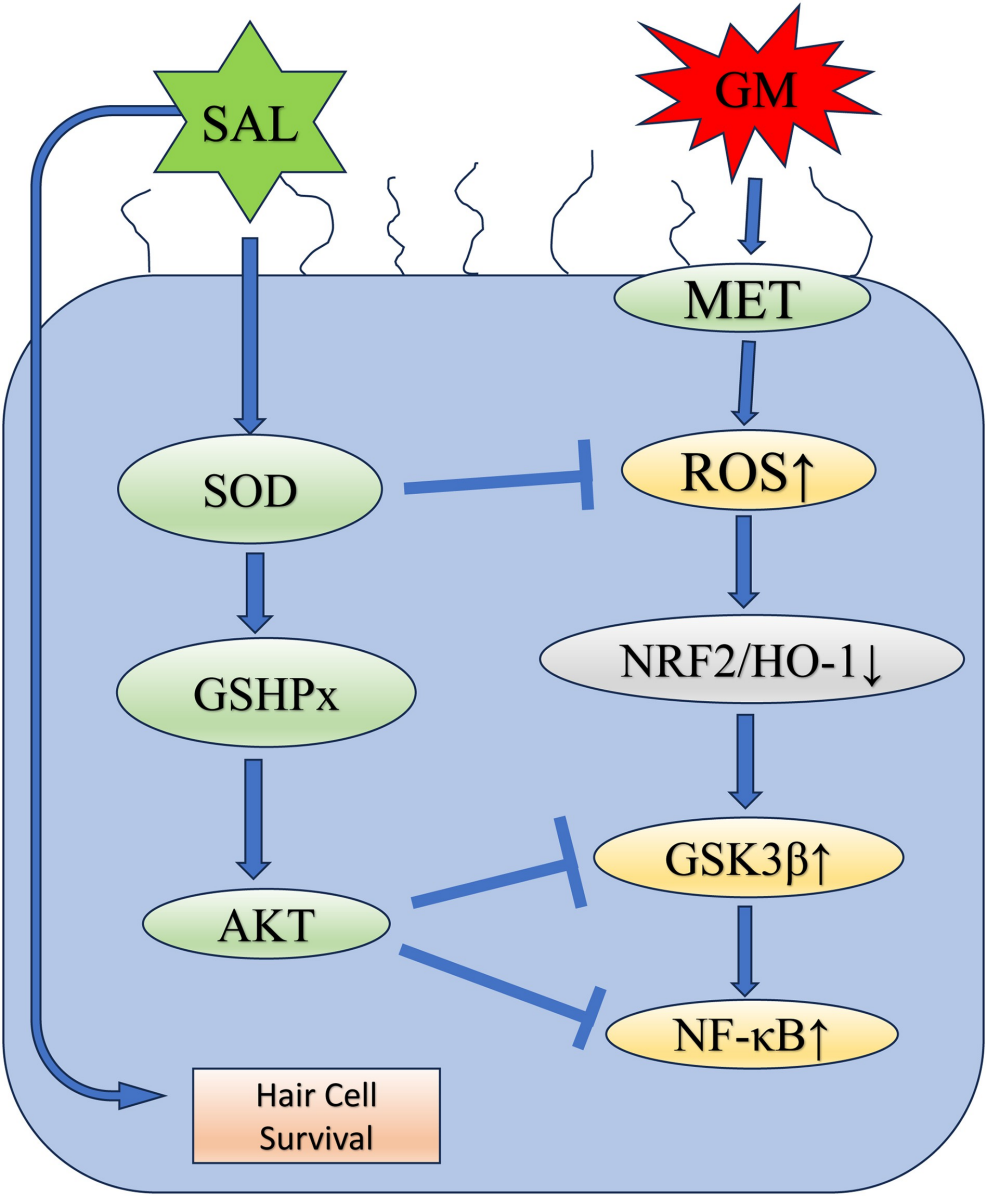
Schematic diagram demonstrated the protective mechanism of SAL in cochlear cultures against GM-induced hair cells injury.

Based upon the experimental results above, we concluded the signaling pathways of GM-induced hearing loss is NRF2/GSK3β/NF-κB and the signaling pathways of SAL protection in GM-induced hearing loss is SOD/GSHPx/AKT (Fig 7).

This study reveals the involvement of the AKT/GSK3β/NRF2 pathway in the oxidative stress induced by GM. Moreover, SAL might affect this pathway to exert its protective effects (Fig 7). The changes were also observed in NF-κB activity (a downstream target of GSK3β). No significant difference in HC loss rate was found compared with the SAL+GM group in the presence of the NF-κB inhibitor, PDTC. SAL can decrease the phosphorylation level of NF-κB, and PDTC combined with SAL had no additive effect. These results indicate that the protective effect of SAL on GM-induced ototoxicity is mainly through direct regulation of NRF2/GSK3β/NF-κB signaling in cochlear tissues.

A limitation of our study is that we used *in vitro* cultured cochlear explants. Although cochlear explant culture is superior to cell line culture, it is still different from *in vivo* conditions. *In vivo* studies are required for further confirm the results and to clarify the specific mechanism underlying the protective of SAL. Further studies are also warranted to investigate the ability of SAL to cross the blood labyrinthine barrier, to determine the route of SAL entry into HCs, to explore whether the protective effect of SAL is specific to GM-induced ototoxicity, to compare the effect of SAL with other anti-oxidative reagents on GM-induced HC damage, and to analyze the effect of SAL on the accumulation of GM in HCs of the inner ear.

In summary, this study demonstrated the anti-oxidant protective mechanism of SAL in cochlear cultures with GM-induced HC injury. The results indicate that SAL exerts a protective effect on HCs against GM-induced injury by inhibiting apoptosis. This protective effect may be achieved by upregulating the NRF2 signaling pathway and inhibiting GSK3β/NF-κB activation. SAL can up-regulate the PI3K/AKT pathway and consequently activate the downstream endogenous anti-oxidant NRF2/HO-1 pathway and suppress GSK3β and NF-κB activation. These results suggest that SAL may be a potential drug for treating cochlear hair cells and provide a potential treatment for clinical application of gentamicin-induced cochlear hair cell damage.

## Supporting information

S1 Data (PDF)
