# Supplementary material for: Therapeutic potential of salidroside in preserving rat cochlea organ of corti from gentamicin-induced injury through modulation of NRF2 signaling and GSK3β/NF-κB pathway
Source: PLoS One. 2024 Mar 14;19(3):e0298529. doi: 10.1371/journal.pone.0298529 (PMC10939193; doi:10.1371/journal.pone.0298529)
Supplement: S1 Data — (PDF) [file pone.0298529.s001.pdf]

Fig1 SAL reduces GM-induced cochlear hair cell loss. A: Cochlear explants were pretreated with 400 μM SAL followed by GM treatment.

Fig1B Changes in cochlear hair cell loss after treatment with different concentrations of SAL（N=3）

OHC missing（%）	Control	100mM	200mM	400mM	800mM
	0.6	0.8	1	1.2	4.4
	1	0.4	0.6	1	3
	1.2	1	0	1	3.6
IHC missing（%）	0.2	0	1.2	1	1.6
	0.8	0.2	0.4	0.2	1.2
	1	1	1	0.4	1.4

FigC-D The percentage of hair cell loss was counted from five selected fields of basal/mid-basal cochlear turns.（N=4）

IHC missing（%）	Control	SAL	GM1	SAL+GM1	GM2	SAL＋GM2
	5	0	10	10	35	10
	0	2.5	12.5	5	21	20
	0	0	5	20	26	15
	0	1	25	5	39	12
OHC missing（%）	Control	SAL	GM1	SAL+GM1	GM2	SAL+GM2
	3	3.2	36.5	12.5	65.7	23.6
	2.8	2.7	41.7	14.7	58.9	12.7
	5	3.5	34.2	23.6	71.4	33.7
	0	0	25.7	8.8	61.4	39.6
	GM1=0.2mM	GM2=0.4mM				

Fig2 Effect of GM and SAL on superoxide dismutase (SOD), GSHPx, and malondialdehyde (MDA) concentrations in cochlear tissues cultured in vitro.

A- C： Changes in SOD, GSHPx and MDA concentrations after GM treatment.

MDA	Con	0.1mM	0.2mM	0.4mM
	5.6	7.9	9.4	15.7
	4.9	5.9	11.5	12.4
	5.3	6.3	11.8	10.6
SOD	Con	0.1mM	0.2mM	0.4mM
	6.5	7.9	3.7	2.6
	6.7	9.6	3.8	1.8
	5.6	5.8	2.7	2.9
GSH-Px	Con	0.1mM	0.2mM	0.4mM
	144	145	83.7	82.6
	106	158	93.8	71.8
	152	131	62.7	92.9

The results of three independent experiments

D-F: D–E: Effect of SAL on SOD, GSHPx and MDA concentrations in the GM-induced ototoxicity model.

Con	GM	SAL	SAL+GM
6.7	15.6	4.6	8.9
5.9	12.8	4.1	7.4
7.1	18.6	6.6	9.8
Con	GM	SAL	SAL+GM
6.5	3.7	7.7	5.6
6.7	2.8	4.8	6.8
7.6	2.7	6.7	4.9
Con	GM	SAL	SAL+GM
144	63.7	163.7	102.6
136	73.8	153.8	121.8
152	52.7	162.7	92.9

The results of three independent experiments

Fig3 Effects of SAL on the mRNA and protein levels of NRF2 and HO-1 in cochlear tissues after GM treatment in vitro.

A: GM administration changed the Nrf2 mRNA level with time.(N=4)

NRF2		Con	SAL	GM	SAL+GM	
	6h	1	1.41	1.73	2.13	
		1	2.08	1.85	1.95	
		1	2.16	1.26	2.57	
		1	1.94	1.65	1.45	
	12h	1	2.69	0.78	2	
		1	1.68	0.63	2.74	
		1	2.17	0.81	1.67	
		1	1.56	0.64	3.04	
	24h	1	1.96	0.61	2.16	
		1	2.25	0.64	1.64	
		1	2.05	0.7	1.95	
		1	1.53	0.63	2.22	

The results of four independent experiments

B: GM administration changed the Ho-1 mRNA level with time.(N=4)

HO-1		Con	SAL	GM	SAL+GM	
	6h	1	2.13	1.33	2.06	
		1	1.08	0.85	1.75	
		1	1.16	1.26	2.47	
		1	1.98	1.35	1.53	
	12h	1	1.69	0.8	2.95	
		1	1.63	0.67	2.7	
		1	2.28	0.86	2.69	
		1	1.39	0.74	3.32	
	24h	1	1.83	0.58	2.35	
		1	1.55	0.65	2.69	
		1	2.15	0.52	1.88	
		1	1.74	0.67	3.41	

The results of four independent experiments

Fig3 Effects of SAL on the mRNA and protein levels of NRF2 and HO-1 in cochlear tissues after GM treatment in vitro.

C:

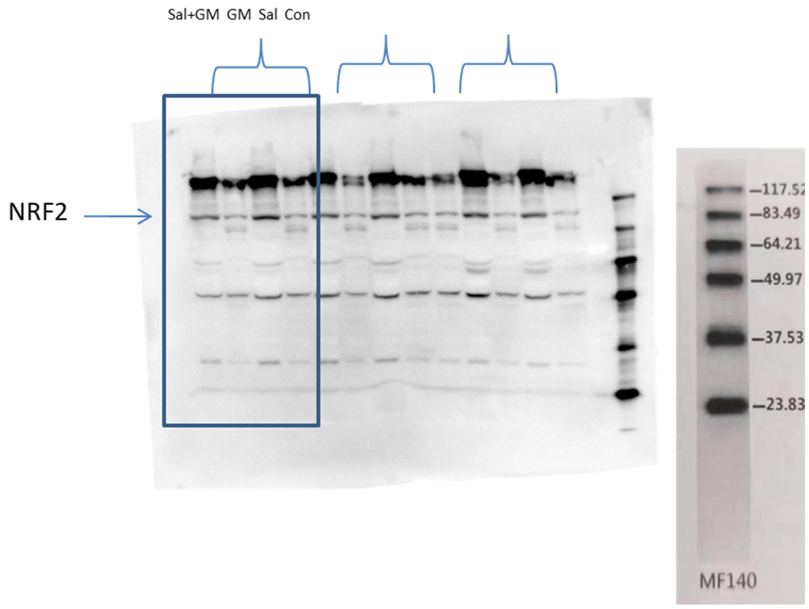

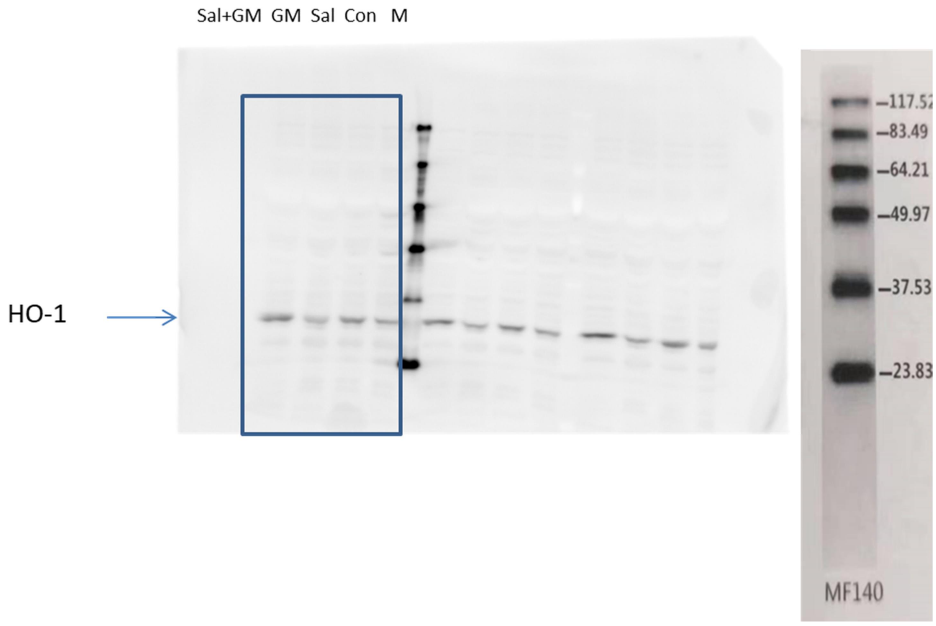

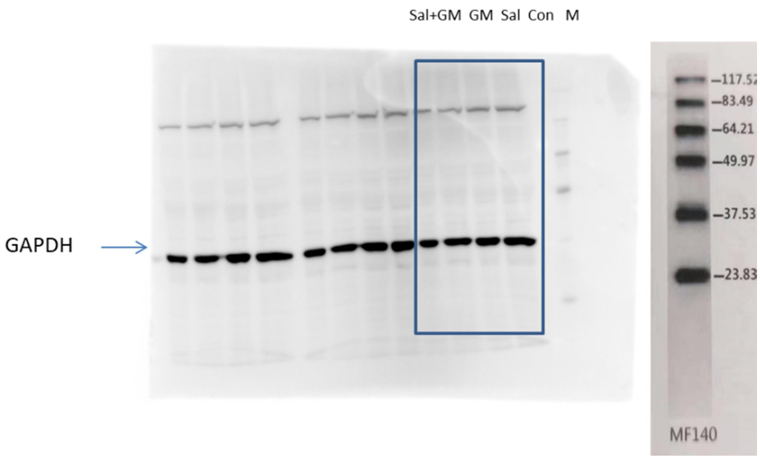

D: Optical density image analysis of NRF2 protein.

NRF2（wb）	Control	SAL	GM	SAL+GM	
	0.179184	0.235948	0.106513	0.384929	
	0.101981	0.299118	0.181282	0.280641	
	0.136402	0.226051	0.158293	0.270977	

The results of three independent experiments

E: Optical density image analysis of HO-1 protein.

HO-1（wb）	Control	SAL	GM	SAL+GM	
	0.22011	0.30974	0.095448	0.621976	
	0.197878	0.268913	0.172897	0.392848	
	0.124815	0.294152	0.130152	0.497424	

The results of three independent experiments

Fig4 Effects of SAL on the protein levels of AKT, GSK3β and caspase 3 in cochlear tissues after GM treatment in vitro.

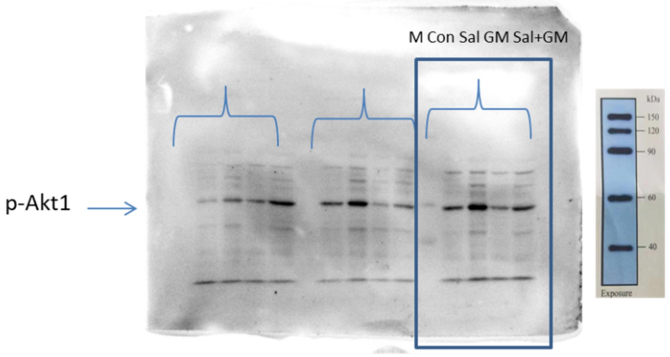

Left：Con, Sal, GM, Sal+GM; Con, Sal, GM, Sal+GM; Marker, Con, Sal, GM, Sal+GM

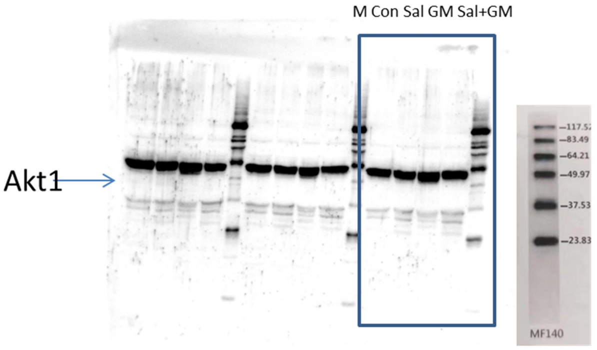

Left：Con, Sal, GM, Sal+GM;Marker, Con, Sal, GM, Sal+GM; Marker, Con, Sal, GM, Sal+GM,marker

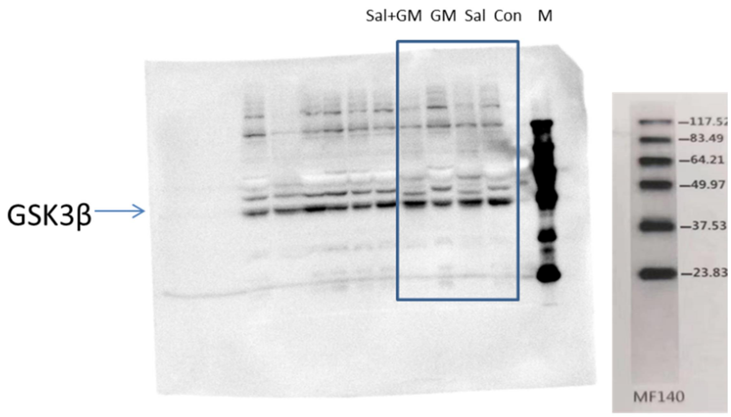

Right:Marker ,Con, Sal, GM, Sal+GM; Con, Sal, GM, Sal+GM;

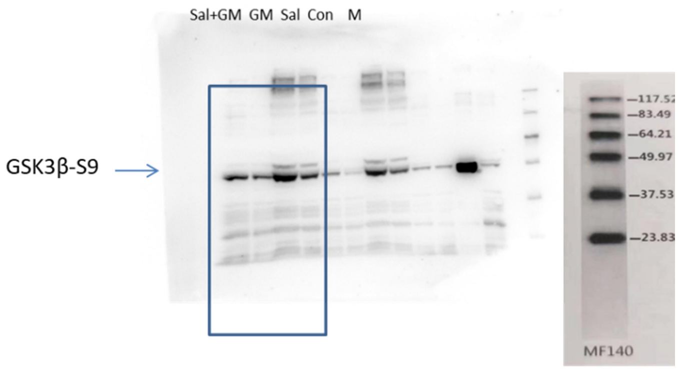

Right:Marker ,Con, Sal, GM, Sal+GM; Con, Sal, GM, Sal+GM;
Con, Sal, GM, Sal+GM

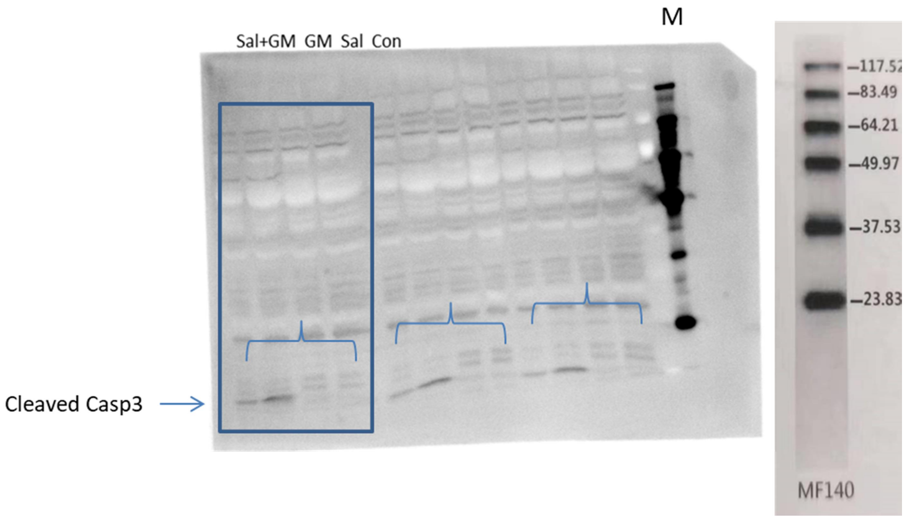

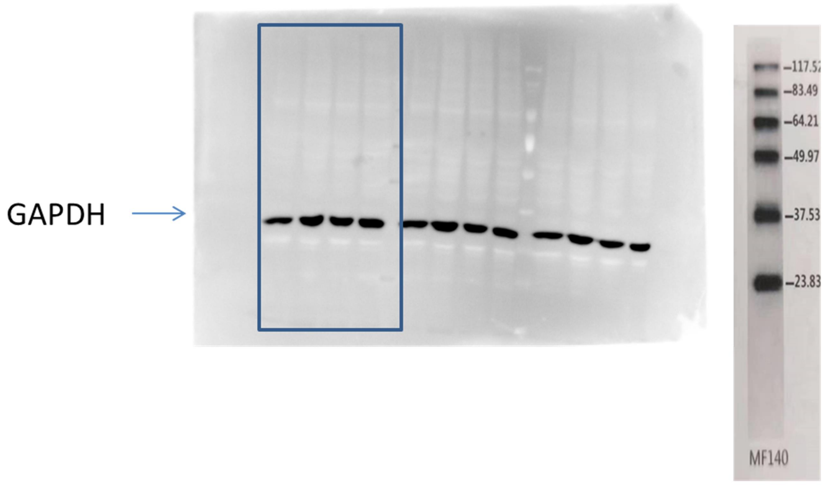

Right:Marker ,Con, Sal, GM, Sal+GM; Con, Sal, GM, Sal+GM;
Con, Sal, GM, Sal+GM

Fig4B The pAKT/AKT ratio was analyzed in different treatment groups using Image J software.

WB					
pakt/akt	Control	SAL	GM	SAL+GM	
	0.09644	0.394374	0.166156	0.293755	
	0.191525	0.444612	0.162654	0.515931	
	0.094221	0.484006	0.275632	0.466066	

Fig4C The pGSK3β/GSK3β ratio was analyzed in different treatment groups

WB					
pGSK/GSK	Control	SAL	GM	SAL+GM	
	0.41	1.04	0.09	0.74	
	0.38	0.67	0.13	0.52	
	0.49	0.68	0.08	0.61	

Figh4E Optical density image analysis of cleaved caspase 3.

		WB					
	cleaved casp3		Control	SAL	GM	SAL+GM	
			0.219995	0.225687	0.498203	0.28495	
			0.098393	0.133316	0.355303	0.211686	
			0.121217	0.123451	0.66449	0.29904	

Figure 5 Cochlear HC loss in the SAL+GM group in response to different signaling pathway inhibitors.

Fig5B　HC survival in each group，n=4

	GM	SA+GM	A+GM+TR	SA+GM+LY	SA+GM+PDTC	SA+GM+AR
HC missing(%)	67.7	23.6	87.9	67.4	34.1	12.7
	78.9	41.7	81.7	77.2	37.8	23.6
	71.4	33.7	75	61.8	27.2	27.5
	61.4	39.6	52.7	64.2	22.4	30.1

Fig6 The expression of AKT, GSK3β, NF-κB and NRF2 in cochlear tissues in the SAL+GM group with or without signaling pathway inhibitors

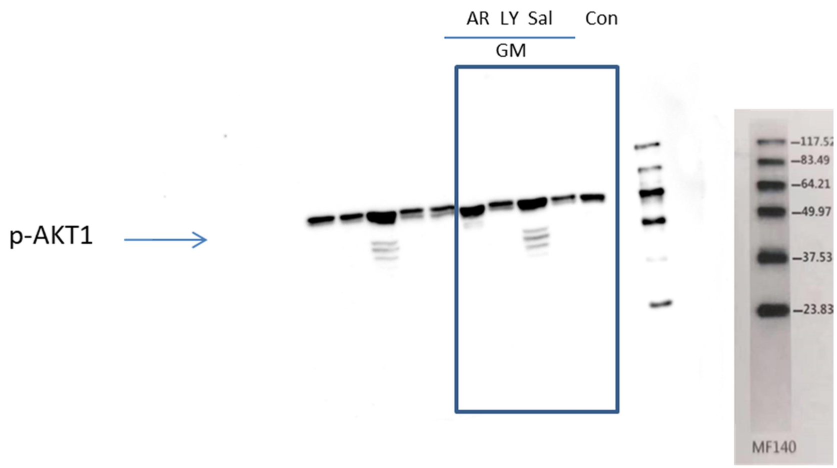

Right: Marker, Con, GM, Sal+GM, LY+GM, AR+GM

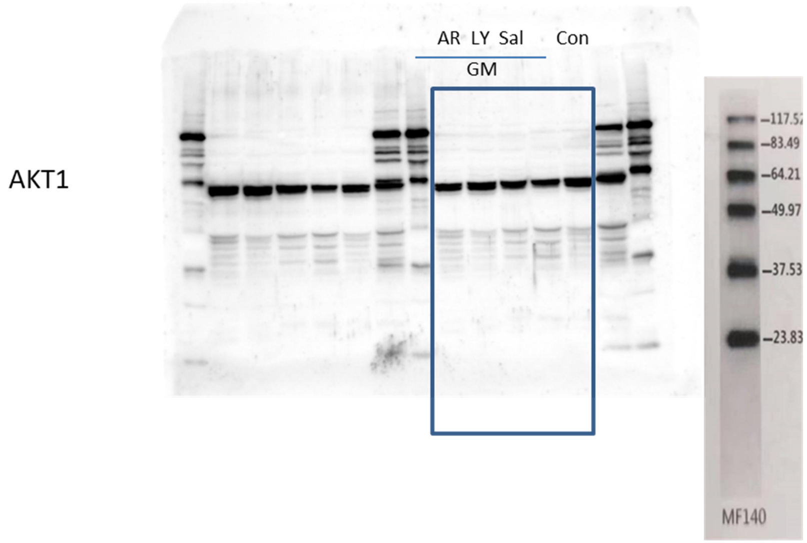

Right: Marker, Con, GM, Sal+GM, LY+GM, AR+GM

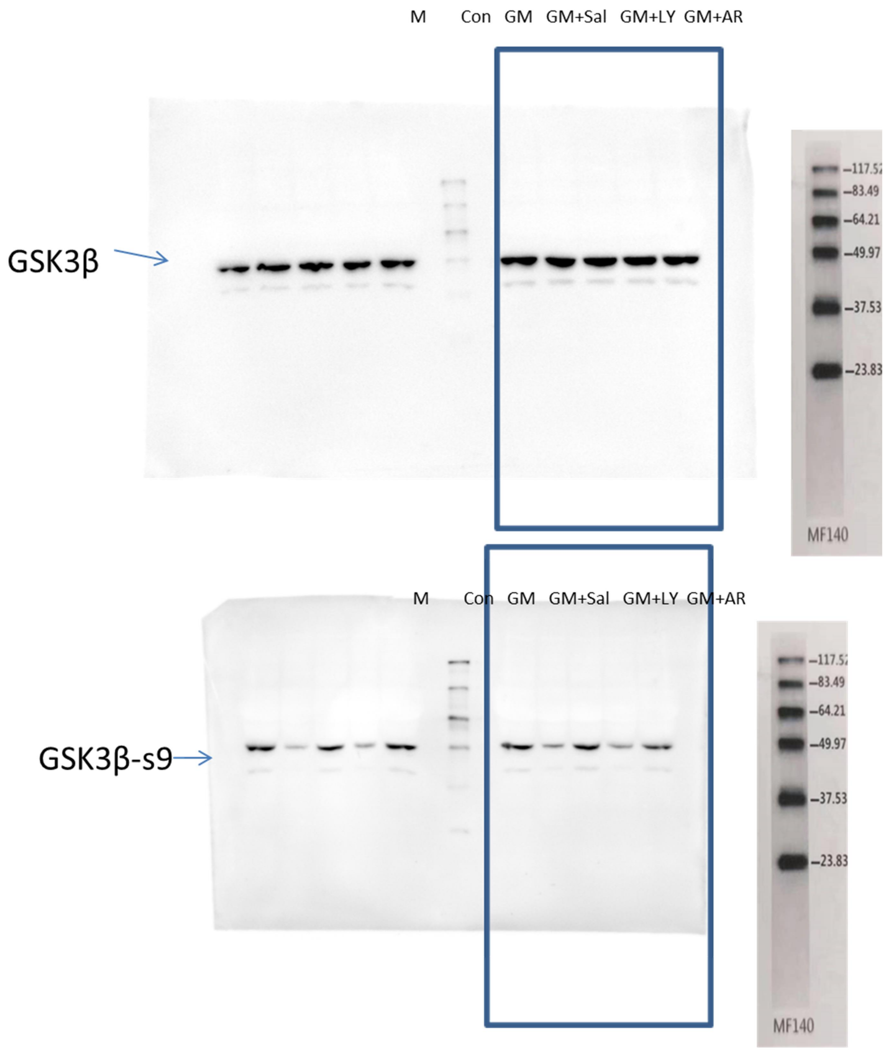

left: Marker, Con, GM, Sal+GM, LY+GM, AR+GM

Con GM GM+Sal GM+LY GM+AR

NRF2

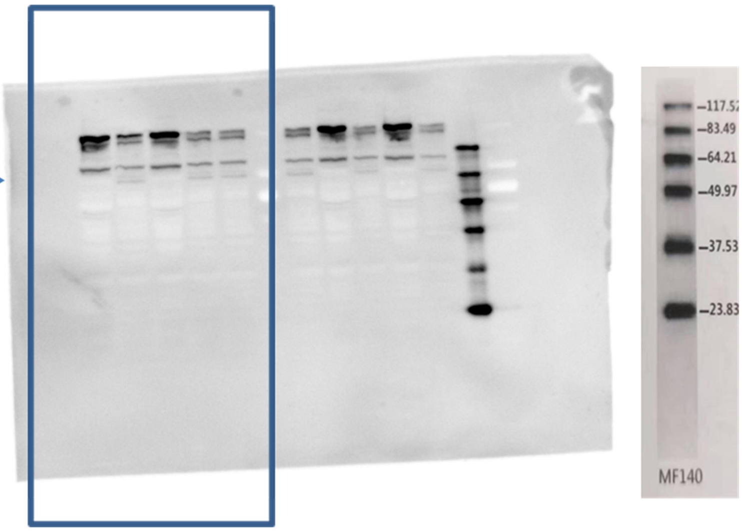

Con GM GM+Sal GM+LY GM+AR

GAPDH

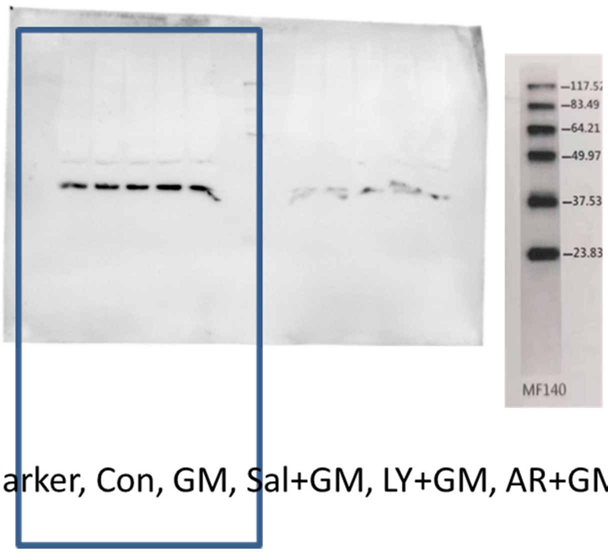

Right: Marker, Con, GM, Sal+GM, LY+GM, AR+GM

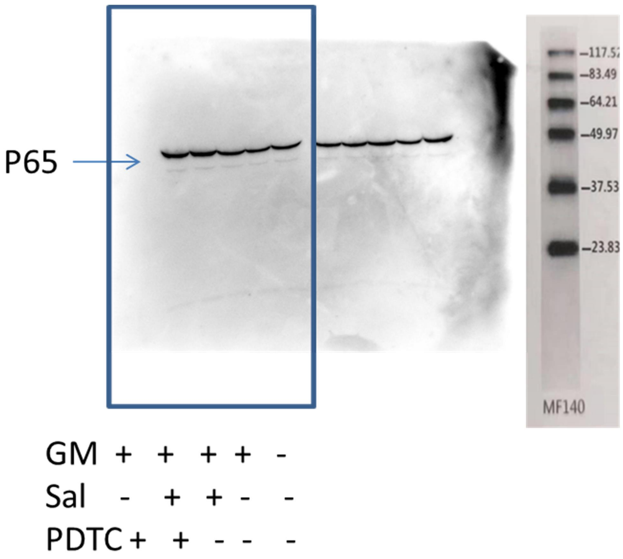
P65
117.5
83.49
64.21
49.97
37.53
23.83
MF140
GM + + + + -
Sal - + + - -
PDTC + + - - -

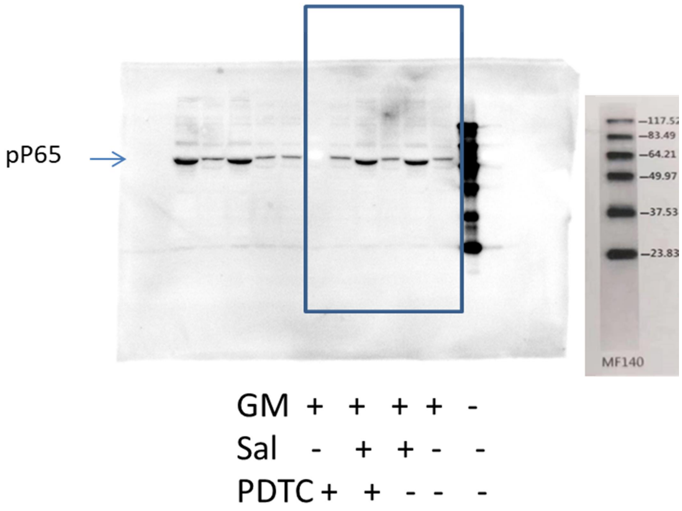
pP65
117.5
83.49
64.21
49.97
37.53
23.83
MF140
GM + + + + -
Sal - + + - -
PDTC + + - - -

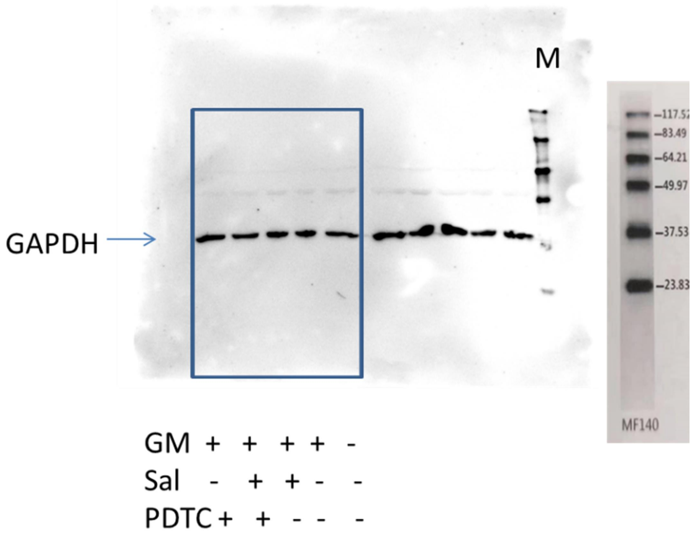

pGSK/GSK	Con	GM	SAL+GM	SAL+GM+LY	SAL+GM+AR	
	0.4487	0.2239	0.6752	0.2632	0.7258	
	0.599942	0.216918	0.701347	0.211761	0.664193	
	0.449499	0.284893	0.727535	0.466383	0.72243	
pAkt/Akt	Con	GM	SAL+GM	SAL+GM+LY	SAL+GM+AR	
	0.838	0.731	1.372	0.784	1.234	
	0.857962	0.849058	1.500492	0.829246	1.217716	
	0.538362	0.667095	1.515708	0.693528	1.011507	
NRF2	Con	GM	SAL+GM	SAL+GM+LY	SAL+GM+AR	
	0.292	0.336	1.023	0.598	1.102	
	0.253	0.275	1.121	0.426	0.887	
	0.324	0.12	0.958	0.485	1.315	
pP65/P65	Con	GM	SAL+GM	AL+GM+PDT	GM+PDTC	
	0.495897	1.376924	0.542711	0.603701	0.410046	
	1.14519	1.768844	0.829346	1.097204	0.351037	
	0.401981	1.467013	0.54527	0.622	0.703285	
